# The Biotin–Avidin Interaction in Biotinylated Gold Nanoparticles and the Modulation of Their Aggregation

**DOI:** 10.3390/nano11061559

**Published:** 2021-06-13

**Authors:** Yanchao Lyu, Álvaro Martínez, Federica D’Incà, Fabrizio Mancin, Paolo Scrimin

**Affiliations:** Department of Chemical Sciences, University of Padova, via Marzolo, 1, 35131 Padova, Italy; yanchao.lyu@studenti.unipd.it (Y.L.); alvmarceb@gmail.com (Á.M.); federica.dinca1@gmail.com (F.D.)

**Keywords:** gold nanoparticles, avidin, biotin, controlled assembly

## Abstract

The biotin–avidin interaction is used as a binding tool for the conjugation of biomolecules for more diverse applications; these include nanoparticle conjugation. Despite this, a thorough investigation on the different aggregates that may result from the interaction of biotinylated nanoparticles (gold nanoparticles, AuNPs, in this work) with avidin has not been carried out so far. In this paper, we address this problem and show the type of aggregates formed under thermodynamic and kinetic control by varying the biotinylated AuNP/avidin ratio and the order of addition of the two partners. The analysis was performed by also addressing the amount of protein able to interact with the AuNPs surface and is fully supported by the TEM images collected for the different samples and the shift of the surface plasmon resonance band. We show that the percentage of saturation depends on the size of the nanoparticles, and larger nanoparticles (19 nm in diameter) manage to accommodate a relatively larger amount of avidins than smaller ones (11 nm). The AuNPs are isolated or form small clusters (mostly dimers or trimers) when a large excess or a very low amount of avidin is present, respectively, or form large clusters at stoichiometric concentration of the protein. Daisy-like systems are formed under kinetic control conditions when nanoparticles first covered with the protein are treated with a second batch of biotinylated ones but devoid of avidin.

## 1. Introduction

**Figure 1:**
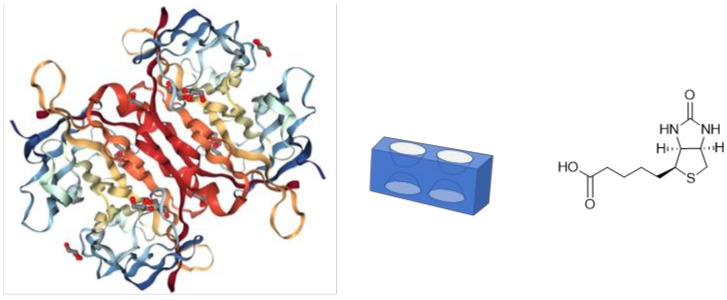
Crystal structure of avidin (**left**), its cartoon representation highlighting the biotin recognition sites on opposite facets (**center**), and chemical structure of biotin (**right**). The structure of the protein (from the Protein Data Bank, code 5CHK) shows the presence of glycerol on the outside, used in the crystallization process.

Biotin (also known as vitamin B7 or vitamin H; see Figure 1) is a small molecule acting as a coenzyme for several carboxylase enzymes. Avidin is a tetrameric glycoprotein with an approximate molecular mass of 66–67 kDa [1]. Not much different from avidin is streptavidin, also a tetrameric protein (not glycosylated), that features 30% sequence identity to avidin, but presents very similar secondary, tertiary, and quaternary structure. The biotin–avidin as well as biotin–streptavidin interactions are among the strongest ones between molecules not relying on the formation of covalent bonds. Each protein can bind up to four biotin molecules, two for each of its facets. When bound, they therefore reside on opposing sites of the protein. The avidin–biotin association constant (K_a_ = 10^15^ M^−1^) is one of the strongest affinities known between biomolecules [2]. For this reason, the avidin/biotin couple is used for many applications where strong interactions between molecules are required without the need to install covalent bonds, like for biomedical applications including the sensing of analytes or the delivery of drugs [3]; it is also very popular in the conjugation of nanosystems [4,5,6]. A search on the Web of Science database using the combined keywords “biotin” and “gold nanoparticles” returned more than 1000 entries. Biotinylated gold nanoparticles are therefore attractive and versatile candidates as universally conjugable agents. However, the multivalent interactions at play enable the formation of complex aggregation patterns. The problem has been addressed under specific conditions or by focusing on how this interaction may be exploited to govern specific targeting [7,8,9,10,11,12,13]. The most accurate investigation carried out so far on this issue was performed by Pérez-Luna using streptavidin and biotinylated nanoparticles [14]. It presented conditions leading to nanoparticle aggregation and pointed out the relevance of slow kinetics in the disaggregation process. Most of the studies aim at exploiting the practical application of the biotin/avidin interaction as in immuno-biosensing [4]; other biological application [5], such as in targeted delivery [6]; or were devoted to control the interaction between different types of gold nanosystems like nanorods [7,8] or nanosystems presenting different geometries [10]. A thorough analysis on how the biotin-(strept)avidin binding may control both the interaction of biotinylated nanosystems with the protein and how this may affect the crosslinking of the system is, however, still well deserved. For this reason, we embarked in a rigorous analysis of the interaction of biotinylated gold nanoparticles (AuNPs), as a model nanosystem, with avidin. Detailed information on the conditions leading to different types of interactions between the nanoparticles have been obtained, both under thermodynamic and kinetic control, and are discussed in this paper.

## 2. Materials and Methods 

### 2.1. General

Chemicals, solvents, avidin, and deuterated solvents for NMR were obtained from Sigma-Aldrich and used as purchased without further purification unless stated otherwise. Ultrapure water was prepared with a Millipore MilliQ^®^ (Merck KGaA, Darmstadt, Germany) system.

TLC were run on 0.2 mm Macherey–Nagel Alugram Xtra SIL G/UV254 (Fisher Scientific Italia, Rodano (MI), Italy) plates, revealed typically under 365 nm irradiation and phosphomolybdic acid ethanolic solution. Terminal alkynes were revealed employing ethanolic KMnO_4_ and free amines with ninhydrin. Molecules containing polyethyleneglycol chains were revealed with the Dragendorff’s reagent [15]. Azides were revealed by an in situ derivatization approach by reducing them with PPh_3_ under mild heating and revealing the resulting primary amine with ninhydrin. 

Column chromatography was performed using silica gel as stationary phase unless otherwise stated. Silica gel used was Macherey–Nagel Keiselgel 60 (Fisher Scientific Italia, Rodano (MI), Italy) with particles sizes of 0.04–0.063 mm (flash) or 0.063–0.2 mm (gravity). 

Hygroscopic reagents were kept inside a desiccator except for tetrachloroauric acid, which was immediately used to prepare a stock solution in water upon arrival. The stock solution was stored frozen at −20 °C.

Glassware in contact with gold nanoparticles was washed with aqua regia before and after its use and rinsed with ultrapure water. All gold nanoparticle preparation and purification were carried out with ultrapure water. Nanoparticles were purified by centrifugation on a Hettich Universal 320 R centrifuge operating with a swinging rotor (V ≤ 15 mL, rpm ≤ 5000) or a 45° fixed angle rotor (V ≤ 5 mL, rpm ≤ 12,000) or an Eppendorf miniSpin Plus (V ≤ 1.5 mL, rpm ≤ 14,500) depending on the sample.

Molecular sieves (4 Å) were activated by heating at 300 °C for 24 h under vacuum and stored under nitrogen before use.

### 2.2. Physical Measurements

UV–Visible spectra were acquired on a Varian Cary 50 or Cary 100 spectrophotometer employing 10 mm path length Hellma Suprasil^®^ quartz cuvettes.

ESI mass spectra were recorded on an Agilent Technologies 1100 Series system equipped with a binary pump (G1312A) and MSD SL Trap mass spectrometer (G2445D SL) with ESI source from solutions in methanol or acetonitrile; in positive mode eluents contained 0.1% formic acid. Mass reported correspond to monocationic protonated adducts.

NMR spectra were recorded on a Bruker Avance DPX 200, Avance 300, or AVIII 500 spectrometers operating at 200, 300, and 500 MHz for ^1^H and 50.3, 75.5, and 125.7 MHz for ^13^C, respectively. ^1^H and ^13^C NMR spectra were calibrated using residual solvent signals, whereas for ^31^P calibration an automatic spectrometer reference was used. Residual solvent signals were assigned according to previously reported values [16]. The spectra are reported in Figures S1–S4 along with Schemes S1 and S2 useful for identifying key protons. 

Thermogravimetric analyses (TGA) were carried out on 0.5–3 mg of nanoparticles with a TA Instruments Q5000 IR instrument. Solvent was removed by heating the sample at 100 °C for 30 min and then a 10 °C/min temperature ramp was applied from 100 to 1000 °C.

Transmission electron microscopy analysis was run on a FEI Tecnai G12 microscope operating at 100 kV, and images were registered with a OSIS Veleta 4K camera. Samples were typically deposited on a copper grid, and the excess of solvent was removed with filtering paper. Stained samples were treated by putting in contact the sample-containing grid with a drop of uranyl acetate solution for 5 min in the absence of light. Size distribution analysis was carried out by modelling nanoparticle intensity profiles employing PEBBLES and size distribution calculated by performing direct statistics on the previous modelled nanoparticles with PEBBLEJUGGLER [17].

### 2.3. Synthesis and Characterization of Chemical Compounds

Zwitterionic thiol **2** [18], pentafluorophenyl ester **3** [19], and PEGaminoazide **4** [20] were prepared by following previously reported procedures.

***N*-(17-azido-3,6,9,12,15-pentaoxaheptadecyl)-5-((3a*S*,4*S*,6a*R*)-2-oxohexahydro-1*H*-thieno[3,4-*d*]imidazol-4-yl)pentanamide (5).** To a solution of d-biotin (139 mg, 0.57 mmol, 1 eq), EDC (218 mg, 1.14 mmol, 2 eq) and DMAP (7 mg, 0.057 mmol, 0.1 eq) in anhydrous DMF (5 mL) at 0 °C, a solution of **4** (174 mg, 0.57 mmol, 1 eq) in anhydrous DMF (5 mL) was added dropwise. The reaction was allowed to warm to room temperature and stirred overnight under N_2_ atmosphere. The solvent was removed under vacuum and the product purified by column chromatography in CH_2_Cl_2_:MeOH 8:1. Yield: 170 mg (56%).

^1^H NMR (500 MHz, Methanol-*d*_4_): δ_H_ (ppm): 4.49 (ddd, 1H, ^3^*J*_HH_ = 7.9, 5.0, 0.9 Hz, Biotin-H_a_), 4.31 (dd, 1H, ^3^*J*_HH_ = 4.5 Hz, Biotin-H_b_), 3.70-3.54 (m, 18H, PEG-OCH_2_), 3.55 (t, 2H, ^3^*J*_HH_ = 5.4 Hz, OC*H*_2_CH_2_NH), 3.40–3.34 (m, 4H, C*H*_2_CH_2_N_3_ + C*H*_2_NH), 3.21 (ddd, 1H, ^3^*J*_HH_ = 8.9, 5.9 Hz, Biotin-H_c_), 2.96–2.89 (m, 1H, Biotin-H_endo_), 2.71 (d, 1H, ^2^*J*_HH_ = 12.7 Hz, Biotin-H_exo_), 2.22 (t, 2H, ^3^*J*_HH_ = 7.5 Hz, CH_2_C(O)), 1.79–1.56 (m, 4H, CH_2_N_3_ + C*H*_2_CH_2_C(O)), 1.50–1.40 (m, 4H, CH_2_C*H*_2_).

ESI‒MS: For C_22_H_41_N_6_O_7_S, observed mass 533.43, calculated 533.28.

***N*-(17-amino-3,6,9,12,15-pentaoxaheptadecyl)-5-((3a*S*,4*S*,6a*R*)-2-oxohexahydro-1*H*-thieno[3,4-*d*]imidazol-4-yl)pentanamide (6).** To a flask containing **5** (300 mg, 0.56 mmol, 1 eq) a THF (4 mL) solution of triphenylphosphine (160 mg, 0.62 mmol, 1.1 eq) and then water (2 mL) were added and the solution vigorously stirred overnight. The organic solvent was removed under vacuum and the aqueous phase washed twice with CH_2_Cl_2_ and once with Et_2_O. The desired product was obtained as a white solid upon freeze-drying. Yield: 240 mg (84%).

^1^H NMR (500 MHz, Methanol-*d*_4_): δ_H_ (ppm): 4.49 (ddd, 1H, ^3^*J*_HH_ = 8.0, 5.0, 0.9 Hz, Biotin-H_a_), 4.31 (dd, 1H, ^3^*J*_HH_ = 4.5 Hz, Biotin-H_b_), 3.68–3.60 (m, 16H, PEG-OCH_2_), 3.60–3.50 (m, 4H, 2 × OC*H*_2_CH_2_N), 3.36 (t, 2H, ^3^*J*_HH_ = 5.5 Hz, C*H*_2_NH), 3.21 (ddd, 1H, ^3^*J*_HH_ = 9.0, 5.8 Hz, Biotin-H_c_), 2.97–2.90 (m, 1H, Biotin-H_endo_), 2.87 (t, 2H, ^3^*J*_HH_ = 5.3 Hz, C*H*_2_CH_2_NH_2_), 2.71 (d, 1H, ^2^*J*_HH_ = 12.7 Hz, Biotin-H_exo_), 2.22 (t, 2H, ^3^*J*_HH_ = 7.4 Hz, CH_2_C(O), 1.81–1.54 (m, 4H, C*H*_2_CH_2_), 1.50–1.40 (m, 2H, CH_2_C*H*_2_).

^13^C{^1^H} NMR (125.7 MHz, Methanol-*d*_4_): δ_C_ (ppm): 176.2 (2 × CO_amide_), 166.1 (CO_biotin_), 72.2, 71.6, 71.5, 71.2 (OCH_2_), 70.6 (*C*H_2_CH_2_NH), 63.4 (Biotin-C_b_), 61.6 (Biotin-C_a_), 57.0 (Biotin-C_c_), 41.8 (CH_2_NH_2_) 41.0 (Biotin-C_d_), 40.3 (CH_2_NH), 36.7 (CH_2_C(O)), 29.8, 29.5 (*C*H_2_CH_2_), 26.8 (*C*H_2_CH_2_C(O)).

ESI‒MS: For C_22_H_43_N_4_O_7_S, observed mass 507.44, calculated 507.29.

*S*-(5,25-dioxo-1-((3a*S*,4*S*,6a*R*)-2-oxohexahydro-1*H*-thieno[3,4-*d*]imidazol-4-yl)-9,12,15,18,21-pentaoxa-6,24-diazadotriacontan-32-yl) ethanethioate (7). A solution of 6 (240 mg, 0.47 mmol, 1 eq) was stirred in the presence of activated molecular sieves in anhydrous DMF (8 mL) for 30 min, then a solution of 3 (269 mg, 0.705 mmol, 1.5 eq) in anhydrous DMF (3 mL) was added, followed by DIPEA (98 µL, 0.56 mmol, 1.2 eq) and the reaction was heated at 70 °C for 2 days. The mixture was filtered, the solvent removed under vacuum, and the product purified by column chromatography in CH_2_Cl_2_:MeOH 10:1 → 6:1. Yield: 190 mg (57%).

^1^H NMR (500 MHz, Methanol-*d*_4_): δ_H_ (ppm): 4.50 (ddd, 1H, ^3^*J*_HH_ = 7.9, 5.0, 0.9 Hz, Biotin-H_a_), 4.31 (dd, 1H, ^3^*J*_HH_ = 4.5 Hz, Biotin-H_b_), 3.68–3.59 (m, 16H, PEG-OCH_2_), 3.54 (m, 4H, 2 × OC*H*_2_CH_2_NH), 3.36 (m, 4H, C*H*_2_NH), 3.21 (ddd, 1H, ^3^*J*_HH_ = 8.9, 5.8 Hz, Biotin-H_c_), 2.93 (dd, 1H, ^2^*J*_HH_ = 12.8 Hz, Biotin-H_endo_), 2.86 (t, 2H, ^3^*J*_HH_ = 7.3 Hz, CH_2_S), 2.71 (d, 1H, Biotin-H_exo_), 2.30 (s, 3H, CH_3_), 2.25-2.16 (m, 4H, CH_2_C(O)), 1.80–1.51 (m, 8H, C*H*_2_CH_2_), 1.49–1.28 (m, 8H, CH_2_C*H*_2_).

^13^C{^1^H} NMR (125.7 MHz, Methanol-*d*_4_): δ_C_ (ppm): 197.6 (CO_thioacetate_), 176.3, 176.1 (2 × CO_amide_), 166.1 (CO_biotin_), 71.6, 71.5 (OCH_2_), 71.3, 70.6 (2 × *C*H_2_CH_2_NH), 63.4 (Biotin-C_b_), 61.6 (Biotin-C_a_), 57.0 (Biotin-C_c_), 41.0 (Biotin-C_d_), 40.3 (2 × CH_2_NH), 37.0, 36.7 (2 × *C*H_2_C(O)), 30.7 (*C*H_2_CH_2_S), 30.5 (CH_3_), 30.1–29.6 (*C*H_2_*C*H_2_), 29.5 (CH_2_S), 26.9, 26.8 (2 × *C*H_2_CH_2_C(O)).

ESI‒MS: For C_32_H_59_N_4_O_9_S_2_, observed mass 707.29, calculated 707.37.

8-Mercapto-*N*-(19-oxo-23-((3a*S*,4*S*,6a*R*)-2-oxohexahydro-1*H*-thieno[3,4-*d*]imidazol-4-yl)-3,6,9,12,15-pentaoxa-18-azatricosyl)octanamide (1). Thioacetate deprotection was carried out by refluxing a solution of the protected form in a 1:1 mixture of 6 M aq. HCl and EtOH for 2–3 h. The solvent was removed under vacuum and the product obtained in quantitative yield.

^1^H NMR (500 MHz, Methanol-*d*_4_): δ_H_ (ppm): 4.59 (ddd, 1H, ^3^*J*_HH_ = 7.9, 5.0, 0.8 Hz, Biotin-H_a_), 4.40 (dd, 1H, ^3^*J*_HH_ = 4.4 Hz, Biotin-H_b_), 3.77-3.61 (m, 16H, PEG-OCH_2_), 3.55 (t, 4H, ^3^*J*_HH_ = 5.5 Hz, 2 × OC*H*_2_CH_2_NH), 3.38 (m, 4H, C*H*_2_NH), 3.29-3.22 (m, 1H, Biotin-H_c_), 2.97 (dd, 1H, ^2^*J*_HH_ = 12.9 Hz, Biotin-H_endo_), 2.76 (d, 1H, Biotin-H_exo_), 2.50 (t, 2H, ^3^*J*_HH_ = 7.3 Hz, CH_2_S), 2.28–2.19 (m, 4H, CH_2_C(O)), 1.80–1.55 (m, 8H, C*H*_2_CH_2_), 1.51–1.28 (m, 8H, CH_2_C*H*_2_).

^13^C{^1^H} NMR (125.7 MHz, Methanol-*d*_4_): δ_C_ (ppm): 176.7, 176.3 (2 × CO_amide_), 165.9 (CO_biotin_), 71.6, 71.5 (OCH_2_), 71.3, 70.4 (2 × *C*H_2_CH_2_NH), 64.0 (Biotin-C_b_), 62.4 (Biotin-C_a_), 56.9 (Biotin-C_c_), 40.8 (Biotin-C_d_), 40.6 (2 × CH_2_NH), 36.8, 36.5 (2 × *C*H_2_C(O)), 35.1 (*C*H_2_CH_2_S), 30.1–29.2 (*C*H_2_*C*H_2_), 27.0, 26.8 (2 × *C*H_2_CH_2_C(O)), 24.9 (CH_2_S).

HR‒MS: For C_30_H_57_N_4_O_8_S_2_, observed mass 665.3625, calculated 665.3618, difference 0.7 mDa.

### 2.4. AuNPs Synthesis and Characterization

Typically, to 5.6 mL of water, was sequentially added under vigorous stirring 1.6 mL of sodium citrate (510 mM), 250 µL of silver nitrate (10 mM), and 500 µL of tetrachloroauric acid (250 mM). The mixture was stirred for 5 min. During this time, the solution changed from the initial yellow color to green, gray, and finally black. Then, the solution was quickly added to 117 mL of boiling water and heated under reflux for 1 h, becoming wine-red after a few seconds. The citrate-capped nanoparticles solution obtained was then allowed to cool down to room temperature, and **2** (freshly prepared from its protected thioacetate) was added in 1–2 mL of isopropanol and left stirring overnight.

Nanoparticles were purified by ultrafiltration using 15, 4, or 0.5 mL Amicon^®^ Ultra centrifuge filters with 100 KDa cut-off (2.5 min at 2000 rpm) or 10 KDa cut-off (5000 rpm for 6 min). Prior to use, filters were prewashed 5 times with 1:1 H_2_O:MeOH, then nanoparticles were filtered and re-diluted 5 times with 1:1 H_2_O:MeOH and 5 times with pure water. Typical concentrated volumes after centrifugation for 15, 4, and 0.5 mL filtering units were 1.5, 0.5, and 0.1 mL, respectively. Purified nanoparticles solutions were frozen in an acetone/dry ice bath and freeze-dried overnight on a Cinquepascal 105PDGT lyophilizer equipped with and Edwards XDS 10 Pump (P ≈ 0.2 mbar, T = −50 °C). Typically, an amount between 22 and 26 mg of functionalized nanoparticles was obtained, being around 75% yield.

Nanoparticle’s core was characterized by TEM analysis (Figures S5–S7). In a typical batch, a minimum of 200 measured nanoparticle sizes were used to calculate the average size and the associated standard deviation. The organic monolayer was studied by thermogravimetric analysis, which provided the amount of thiol (organic fraction) per mass unit of AuNPs (see Supporting Information Figures S8–S10). Depending on the size of the gold core, the concentration of thiols on the monolayer varied in the range from 0.1 to 0.3 µmols of thiol per mg of nanoparticle. Nanoparticles were analyzed by solution 1 (NMR) to ensure the absence of free thiol in solution (no sharp signals present, see Figure S11).

### 2.5. Thiol Exchange Protocol

Nanoparticles were dissolved in methanol, and the desired thiol was added in methanol after being freshly deprotected. Final solution was typically 1.5 mM in each thiol (ratio 1:1) and was allowed to exchange at 30 °C for 18 h. Subsequently the reaction was diluted with water, concentrated and re-diluted 5 times with 1:1 H_2_O:MeOH, 5 times with pure water by using Amicon^®^ Ultra filters with 100 KDa cut-off and finally frozen with acetone/dry ice and freeze-dried overnight. The extent of the exchange was determined by ^1^H-NMR spectroscopy after thiols etching from the nanoparticles surface performed by treating 500 µL of nanoparticles solution in methanol-*d*_4_ with 100 µL of a saturated solution of iodine in methanol-*d*_4_. Typically, around 3 mg of AuNPs were employed, resulting in a final concentration of thiols around 1 mM.

### 2.6. Assessment of Biotinylated AuNPs—Avidin Interaction

The gold nanoparticles were dissolved in buffer (HEPES 10 mM pH 7.3, 150 mM NaCl, 10 mM MgCl_2_) to reach a concentration of 0.0238 mg/mL and 4 equivalents of avidin (with respect to the active biotin thiols on the nanoparticles) were added and the solution left stirring for 1 h. The excess of avidin was removed by filtration and re-dilution with buffer, using Amicon^®^ Ultra filters with 100 kDa cut-off, for 5 times. In a fluorescence cuvette, the avidin covered gold nanoparticles are set to 0.8 absorbance at 520 nm and then the dye (Atto 565, stock solution 0.29 mM in DMSO) is added directly to the cuvette. The cuvette is shaken, allowed to incubate for 10 min and the fluorescence measured at the emission wavelength (λ_ex_ = 565 nm, λ_em_ = 590 nm).

## 3. Results and Discussion

### 3.1. Synthesis and Characterization of Biotinylated AuNPs

**Scheme 1:**
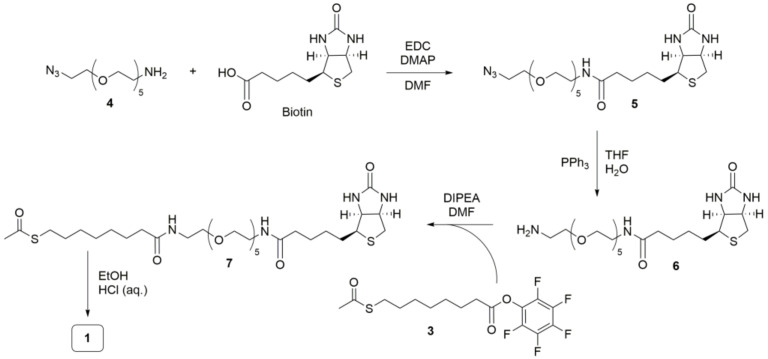
Synthetic scheme followed for the preparation of thiol **1**.

For our investigation we have prepared gold nanoparticles of 9–19 nm in diameter passivated with the biotinylated thiol **1** and the zwitterionic thiol **2** chosen for the reported low affinity of proteins for zwitterionic nanoparticles [21]. Thus, the AuNPs we have used in our experiments are constituted by a mixed monolayer of an “inert” thiol (**2**) and the “active” one (**1**). Biotinylated thiol **1** was obtained following the synthetic route depicted in Scheme 1. The synthetic steps follow consolidated procedures and were all characterized by good to high yields as reported in the Materials and Methods section.

Thiol **1** possesses a linker comprising a hydrocarbon moiety close to the thiol and an oligoethylene glycol moiety (composed by 5 oxyethylene units) close to the biotin. In our experience, a hydrophobic region close to the AuNPs surface ensures higher stability to them by preventing interactions with the aqueous solvent [22,23]. The oxyethylene units were designed to allow both the hydration of the surface of the monolayer and the conformational freedom of the biotins [24]. Both properties could, in principle, affect the binding to the protein.

The AuNPs were prepared following an improved Turkevich protocol as reported by Xia et al. [25] with minor modifications introduced by us to increase the yield and minimize size dispersion [26]. The procedure relies on the reduction of minimum amounts of Ag(I) that generate the seeds for the growth of the AuNPs with relatively low size dispersion. The key role of seeds in the homogeneous growth of AuNPs prepared following Turkevich’s protocol has been addressed by Polte et al. [27]. Thiol **2** was added to the as-prepared AuNPs using a 10% excess with respect to the calculated amount required to fully cover the surface estimated by dividing the nanoparticle surface by the thiol footprint (see below). The thiol-passivated nanoparticles were then washed with 1:1 methanol/water solutions using a filter with cut-off 100 KDa and eventually with pure water. The ^1^H-NMR spectra of the AuNPs after lyophilization and redissolution in CD_3_OD showed no presence of unbound organic molecules (see Figure S11). The final biotinylated AuNPs were obtained by thiol exchange starting with **2**-passivated nanoparticles and using **1** as entering thiol. At 30 °C and after 18 h of exchange time by using one equivalent of entering thiol with respect to **2**, instead of obtaining AuNPs with a 50:50 ratio of the thiols, we never managed to obtain more than 14% of **1**, as shown in Table 1. The size of the nanoparticle does not appear to affect the extent of the exchange. Nevertheless, as we will see below, the obtained final amount of biotin units present on the AuNPs surface is sufficient to maximize the interaction with the protein. The actual ratio between the two thiols was obtained by treating the nanoparticles with iodine to fully detach them from the gold surface and integrating the ^1^H-NMR of key signals of the disulfide mixture present in solution (Figures S12–S14). The total amount of organic material attached to the gold core was determined by TGA while the AuNPs size (diameter of the gold core) was determined by TEM. To determine the average molecular formula of the biotinylated nanoparticles (see Table 1) we used these data and the gold atomic density of the gold core (59 atoms per nm^3^) [28]. The thiol fingerprint calculated was ca. 0.2 nm^2^ in our case (5 thiols/nm^2^), in good agreement with the reported data for thiols of similar length [29,30]. 

### 3.2. Biotinylated AuNPs Binding to Avidin

**Figure 2:**
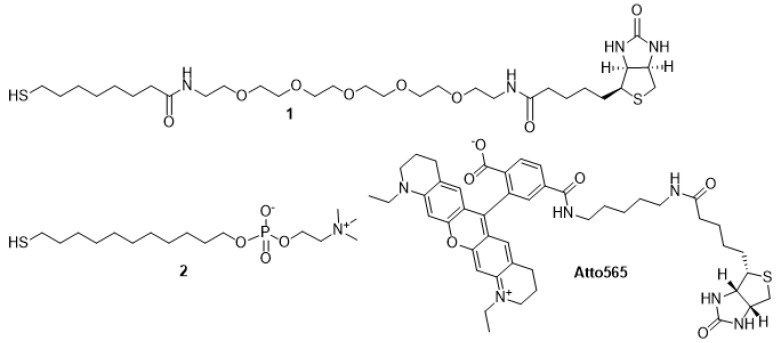
Structure of thiols **1**–**2** used for the passivation of the AuNPs and of the fluorescent biotinylated dye Atto565.

**Figure 3:**
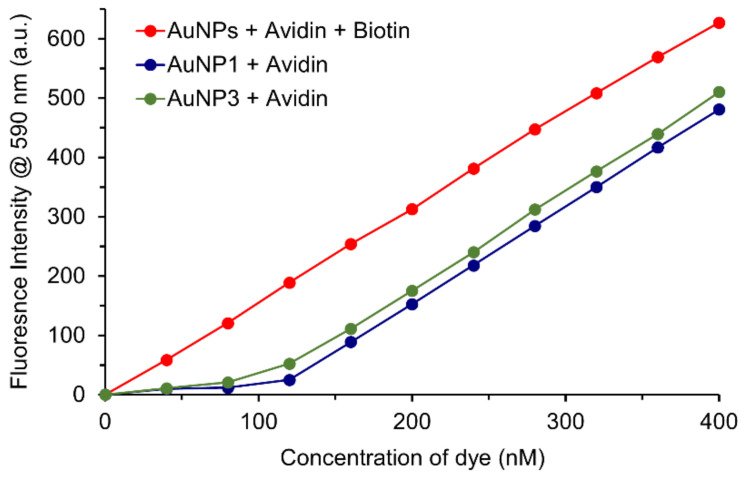
Results of the fluoresce titration experiments for AuNP1 and AuNP3. Control experiments in the present of biotin yielded identical data, only one set is shown for clarity. All AuNPs solutions set at 0.8 absorbance at 520 nm. Relevant results are also reported in Table S1.

In order to assess the number of proteins bound to each AuNP, we performed fluorescence titration experiments using AuNP1 and AuNP3 and the biotinylated dye Atto565 (Figure 2). We have chosen this dye, which is characterized by λ_ex_ = 565 nm and λ_em_ = 590 nm, because these two wavelengths are off the absorbance regions of the AuNPs and no interference was observed also with their SPR band. Notably, we could not use the typical absorbance-based protocol that takes advantage of the change of absorbance of the dye 4’-hydroxyazobenzene-2-carboxylic acid (HABA) because when it is bound to the protein it absorbs at 500 nm, too close to the maximum of the SPR band of the nanoparticles [31]. The florescence experiments rely on the well-known quenching of the fluorescence of molecules when in close proximity to a gold nanoparticle [32]. The nanoparticles were first pre-saturated with avidin and the excess of the protein was removed. Subsequently, we added to the AuNPs solution incremental amounts of the dye. No increase of fluorescence was observed at the beginning because of its quenching due to the binding of the dye to the avidin saturating the nanoparticle surface. This process occurs at the binding sites present on the opposite facet of the protein with respect to those already bound to the biotinylated nanoparticle. Afterwards the fluorescence intensity increased linearly, indicating the dye was no longer able to bind to the nanoparticles and was free in solution. Intersection between these two lines allowed us to determine the saturation concentration of the dye, equivalent to the concentration of free binding sites of the protein (Figure 3). As a control, to rule out non-specific binding events we carried out the experiment after saturation of the binding sites of avidin with an excess of biotin. In that case we expected to observe no quenching of the biotinylated fluorophore and only a linear increase of the fluorescence intensity as was indeed the case (Figure 3).

Using AuNPs solutions with A_520_ = 0.8, the saturation concentration resulted to be 106 ± 2 nM and 94 ± 3 nM (average of three independent experiments) for AuNP1 and AuNP3, respectively. It is reasonable to assume that two molecules of the fluorescent dye bind to each avidin unit, assuming that only one facet of the protein will be exposed to the surface of the nanoparticle, considering its shape (see Figure 1) [1,33,34]. This binding mode is also the one considered in the literature by all authors who have analyzed the interaction biotin/avidin with nanoparticles [8,14,35]. On the basis of the extinction coefficients of the SPR band reported in the literature [34] the concentration of the nanoparticles was 4.6 nM (AuNP1) and 0.99 nM (AuNP3). This translates into 12 and 48 avidins per each AuNP1 and AuNP3, respectively (see Table S1). By dividing the above numbers by the numbers of biotinylated thiols present in the two nanoparticles of different size (as determined by NMR and reported in Table 1) it means that only ~10% of them are involved in the binding of the protein. We have not assessed the dependence of binding of avidin on the percentage of functionalization of the nanoparticle surface. Apart the steric arguments, one may argue that the binding requires the proper placement of the biotins on the monolayer to allow for the optimal geometry for the interaction with two avidins to be achieved. However, as the thiols are relatively free to move within the monolayer [36], such a geometry could be reached with ease after the first binding event has occurred. One might therefore speculate that a large excess of biotin on the surface (like in our case) is not necessary to permit the binding of the maximum possible number of proteins which is dictated by the size of avidin. When considering the average size of the gold nanoparticles and the reported size of avidin (5.6 × 5.0 × 4.0 nm) [33] one can assemble no more than ~29 and ~65 proteins ~11 and ~19 nm nanoparticles, respectively (allowing for the organic monolayer passivating the gold core). This corresponds to ~43% and ~74% of the maximum coverage for AuNP1 and AuNP3, respectively. It appears that the less curved the surface is, the better the proteins assemble on the surface of the nanoparticle approaching the maximum allowed for the size of avidin with 19 nm AuNP3.

#### TEM Micrography

**Figure 4:**
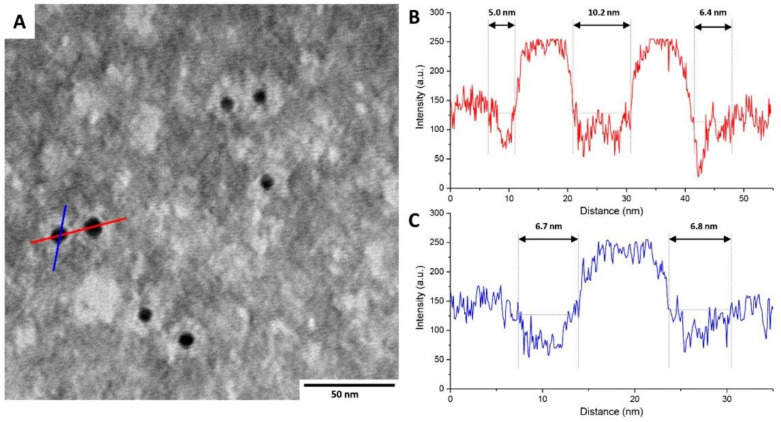
(**A**) TEM image of AuNP2 fully passivated with avidin stained with uranyl acetate. The protein corona is clearly visible as a lighter ring surrounding the dark nanoparticles. (**B**,**C**) Color intensity analysis across the red (**B**) and blue (**C**) lines of TEM image on the left.

The avidin corona surrounding each AuNP can be seen in the TEM images of solutions of nanoparticles fully saturated with the protein (Figure 4A). This technique has been used to follow the interaction of proteins with nanoparticles [37]. Visualization of avidin requires staining with heavy metals to increase the contrast and better visualize the protein. Stained samples showed a low intensity ring around each nanoparticle in contrast with the dark background. These were observed consistently around all nanoparticles present. By analyzing the shade intensity, it was possible to determine the thickness of the protein corona to be within 5–6 nm in full accord with the reported dimensions of avidin (Figure 4B,C) [33]. It is reasonable to think that the “soft” nature of the passivating monolayer does not significantly alter the conformation of avidin once bound to the nanoparticle with one of its larger surfaces where the recognition sites for biotin are located. Note, however, that the aspecific binding of small proteins or peptides to the surface of naked nanoparticles may alter their conformation and orientation [38]. Additional TEM images can be found in the Supporting Information (Figure S15).

### 3.3. Avidin Modulation of Biotinylated AuNPs Crosslinking

**Figure 5:**
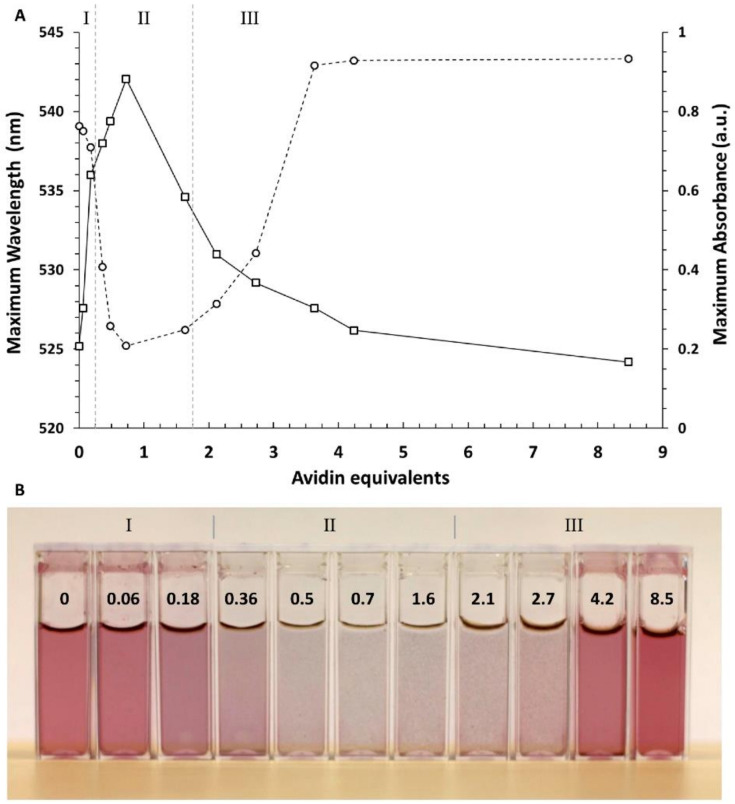
(**A**) Position of the maximum of the SPR band (left axis, squares and solid line) and its absorbance intensity (right axis, circles and dotted line) of AuNP2 upon addition of increasing amounts of avidin; (**B**) The change of color of selected cuvettes following the addition of avidin (equivalents shown on each cuvette, values above 0.36 have been rounded for clarity). Each cuvette represents an independent experiment. Notice the presence of some precipitate in samples 0.36–2.7 (see text for details).

One of the most studied aspects of the interaction between biotinylated nanoparticles and avidin (or streptavidin) is the possibility to modulate the interaction between the nanoparticles in a controlled way [14,35]. The aggregates formed depend strongly on the relative ratio between the protein and the biotin units present on the surface of the nanoparticles. Having assessed with precision not only the number of avidin units present on the AuNPs surface, but also the exact number actually taking part in the binding process, we are in the unique position of precisely controlling this ratio. We are not discovering anything new but simply determining with unprecedented precision how the relative amount of protein affects aggregation. AuNPs of the size we are using in this work allow to follow the aggregation processes spectrophotometrically because the SPR band is not only sensitive to the interaction with the protein but also to the occurrence of aggregation phenomena. Figure 5A reports the dependence of the position of the SPR band and its intensity from the number of equivalents of avidin added to the solution of biotinylated AuNPs. These values represent the maximum of the bands resulting from the summation of the contribution of the SPR band of the isolated nanoparticles and that of the aggregated ones (when present). The spectrum of each sample is reported in Figure S16. One equivalent represents the amount of avidin required to fully saturate its four binding sites, considering only the percentage of biotin units on the surface of the nanoparticles available for binding. Each point in this graph represents an independent experiment since a regular titration cannot be performed. In fact, once the specific equilibrium related to the given amount of avidin added is reached, the shift towards a new equilibrium is a very long process that may require several days. The graph can be divided into three regions (vertical dashed lines of Figure 5A). Region I correspond to a situation in which very little amount of protein has been added (well below one equivalent). Region II corresponds to additions around the stoichiometric amount of protein, while Region III corresponds to the addition of an excess of it. Figure 5B reports the picture of the solutions corresponding to different equivalents of avidin added. The faded color of cuvettes 5–8 is the result of precipitation due to extensive avidin-mediated crosslinking of the nanoparticles resulting in the formation of large aggregates (see Figure S17 for comparison when allowed to deposit).

**Figure 6:**
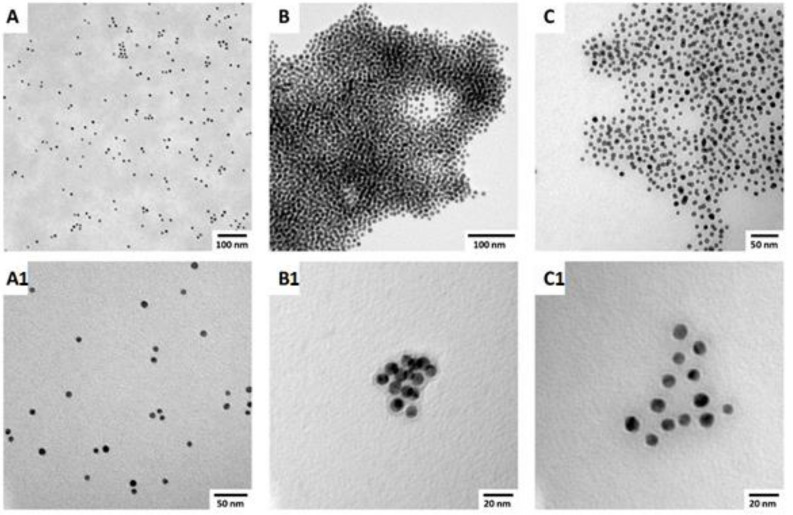
TEM image of AuNP2 recorded after addition of different amounts of avidin. (**A**) 0.18 equivalents (region I of Figure 5A); (**B**) 0.7 equivalents (region II of Figure 5A); (**C**) 8.5 equivalents (region III of Figure 5A); For each condition, two different magnifications are shown (top and bottom, denoted as **A1**–**C1**, the magnified one).

Figure 6A–C reports the TEM images of samples corresponding to each of three regions shown in Figure 5A. The sample of Figure 6A corresponds to the situation in which sub-stoichiometric amounts of protein have been added (Region I). The SPR band has slightly shifted to longer wavelengths, and the TEM image shows the appearance of AuNPs dimers and trimers. Under these conditions the amount of avidin is so little that there is less than one per nanoparticle. The AuNPs in large excess bind to all available binding sites of the protein present, leading to the small aggregates observed. The formation of nanoparticle dimers has been recently reported by Rant et al. [39] under similar conditions. Region II corresponds to a situation in which large clusters form. The shift of the SPR band is more relevant and indicative of the formation of globular aggregates. Theoretically the maximum crosslinking should be reached with one equivalent of nanoparticles, which indeed corresponds to the maximum shift of the SPR band. This extended crosslinking leads also to precipitation. As the amount of avidin increases beyond 1 equivalent, these extended clusters decrease in size until the excess of avidin is such that each nanoparticle is fully covered by the protein (Region III) and no precipitate is formed any more. The protein corona does not affect the position of the SPR band, but it does affect its intensity that increases by ~20%. When the same amounts of avidin were added to non-biotinylated NPs, covered only with **2**, no significant change was observed (see Figure S18).

### 3.4. Kinetic Control of the Cluster Formation

**Figure 7:**
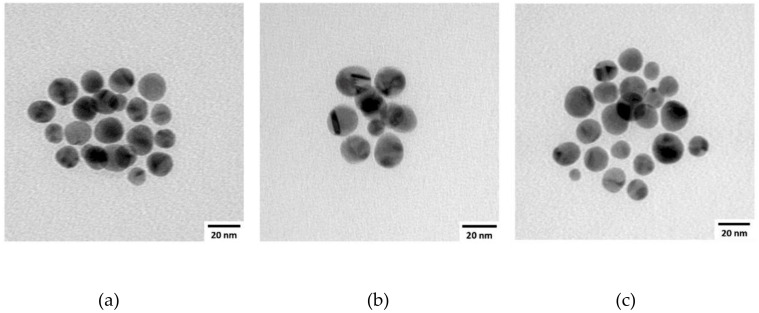
TEM images of three different aggregates of AuNP3 after passivation of their surface with avidin followed by addition of an excess of biotinylated nanoparticles. The cluster of nanoparticles surrounding a central one is clearly visible. Conditions for the experiment: (**a**) a solution of AuNP3 is treated with a large excess (1:8) of avidin that saturates their surface; (**b**) the unbound protein is then removed through three centrifugation/washing cycles; (**c**) a fresh solution of AuNP3 is added (final ratio AuNP3/avidin is 8:1) and the TEM images are collected.

What is described in Figure 5 and Figure 6 above and in Section 3.3. represents the thermodynamic equilibrium [40,41] obtained by mixing biotinylated AuNPs and avidin in different ratios. However, it is possible to induce the formation of different clusters under conditions that are controlled by kinetics and not thermodynamics. We have already shown that AuNPs fully covered by avidin still leave half of the binding sites of the protein free. This occurs when the biotinylated nanoparticles are treated with an excess of protein. We argued that if the excess of avidin is removed and a fresh solution of biotinylated AuNPs is added, binding should immediately occur between the biotin units on the surface and the empty binding sites of the protein bound to the original nanoparticle solution. We proved that this was indeed the case. Thus, a solution of AuNP3 was treated with a large excess of avidin (1:8). The solution remained pink as in the last cuvette on the right of Figure 5B because no aggregation had occurred. Subsequently, the excess avidin was removed through three centrifugation/washing cycles and to the nanoparticle solution an excess of AuNP3 was added (final ratio 8:1). The solution became slightly bluish, but no precipitation was observed. TEM images of the nanoparticles taken from this solution (Figure 7) revealed the formation of small, “daisy-like” clusters characterized by a AuNP surrounded by a corona of AuNPs. Our explanation is that the central nanoparticle derives from the original solution in which it had been fully covered with the protein. The surrounding ones come from the subsequent addition of AuNP3 that bind to the external sites of avidin bound on the original ones. 

From the point of view of the relative ratio of avidin/AuNP3 present in the solution, providing the TEM images of Figure 7, it corresponds to region I of Figure 5 because there is a sub-stoichiometric amount of avidin as a large excess of AuNP3 has been eventually added. However, the obtained aggregates are quite different from the small dimers or trimers obtained under conditions in which the nanoparticles and avidin are mixed in a single shot at that relative ratio (as in Figure 6A). This aggregation mode takes advantage of the very slow response to the change of the relative concentration of AuNPs and avidin once the initial interaction has occurred. This means that by careful additions of precise amounts of nanoparticles and avidin in the correct sequence, one may reach the formation of aggregates under kinetic control that are in fact out of equilibrium [42]. The equilibrium is represented by the small clusters (2–3 nanoparticles) present in Figure 6A. Thus, Figure 7 represents a situation which is out of equilibrium or, said with different words, a kinetic trap. Perez-Luna et al. have shown that the shift of the equilibrium by changing the relative ratio of biotinylated gold nanoparticles and streptavidin may require up to 80 days [14]. This is the obvious consequence of the very high binding constant between the protein and biotin.

## 4. Conclusions

In this paper, we have analyzed in detail the interactions occurring between biotinylated gold nanoparticles of different sizes and avidin. By determining with precision the number of biotin units present on the surface of the passivated gold nanoclusters, we determined how many of them are actually involved in the interaction with protein and how many proteins cover the nanoparticle surface under saturation conditions. We showed that the percentage of saturation depends on the size of the nanoparticles and larger nanoparticles manage to accommodate a relatively larger amount of avidins than smaller ones. The precise assessment of the stoichiometry of the interaction between biotinylated nanoparticles and avidin allowed us to control the aggregation induced by the protein depending on the relative ratio nanoparticles/protein. Taking advantage of the slow kinetic response of the aggregates once formed we were also able to obtain “daisy-like” aggregates that are out of equilibrium from the thermodynamic point of view. We believe that our study will constitute a reference for those who will use the biotin/avidin interaction with nanoparticles to control their aggregation and the targeting of biomolecules.

## Figures and Tables

**Table 1:** Monolayer composition of AuNPs after thiol exchange with biotinylated thiol **1** employed in this work.

Sample	AuNP Diameter ^a^ (nm)	% Biotin Thiol in Monolayer ^b,c^	Estimated Average AuNP Formula
**AuNP1**	11.7 ± 1.7	11.5%	Au_49k_(SR)_2150_
**AuNP2**	8.9 ± 0.8	13.5%	Au_21k_(SR)_1244_
**AuNP3**	18.6 ± 3.1	13.8%	Au_200k_(SR)_5434_

^a^ Determined by TEM analysis; ^b^ The **1**:**2** ratio at the beginning of the exchange experiments was in all cases 1:1; ^c^ Determined by ^1^H-NMR after thiols etching from the gold surface.

## Supplementary Materials

The following are available online at https://www.mdpi.com/article/10.3390/nano11061559/s1, NMR spectra of organic compounds 5, 6, 7, 1 (Figures S1–S4, respectively and Schemes 1 and 2: for the identification of key protons). TEM images and histograms of the NPs 1, 2, 3 (Figures S5–S7, respectively). Thermogravimetric analyses of AuNPs 1, 2, 3 (Figures S8–S10, respectively). ^1^H-NMR spectra of nanoparticles 1, 2, 3 (Figures S11–S14, respectively). TEM images of the protein corona (Figure S15). Spectra in the SPR region (Figures S16). And pictures of the cuvettes under different conditions (Figures S17 and S18), Table S1, reporting the results of the fluorescence titration experiments.
