# Supplementary material for: The Biotin–Avidin Interaction in Biotinylated Gold Nanoparticles and the Modulation of Their Aggregation
Source: Nanomaterials (Basel). 2021 Jun 13;11(6):1559. doi: 10.3390/nano11061559 (PMC8231960; doi:10.3390/nano11061559)
Supplement: Supplementary file 1 [file nanomaterials-11-01559-s001.zip › nanomaterials-1194865-supplementary.pdf]

SUPPORTING INFORMATION

for

The Biotin-Avidin interaction in Biotinylated Gold Nanoparticles and the Modulation of their Aggregation

Yanchao Lyu, Álvaro Martínez, Federica D'Incà, Fabrizio Mancin* and Paolo Scrimin*

Department of Chemical Sciences, University of Padova, via Marzolo, 1, 35131 Padova, Italy

Content

1. Characterization of Chemical Compounds **S2**

2. Characterization of AuNPs **S6**

3. Quantification of Thiol Exchange **S9**

4. Assessment of Biotinylated AuNPs-Avidin Interaction **S11**

5. Avidin-AuNPs Crosslinking **S12**

1. Characterization of Chemical Compounds

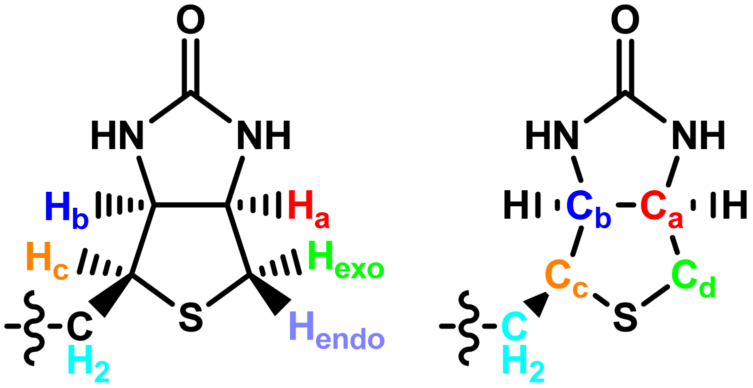

Scheme S1. Notation employed when reporting biotin ^{1}H and ^{13}C-NMR signals.

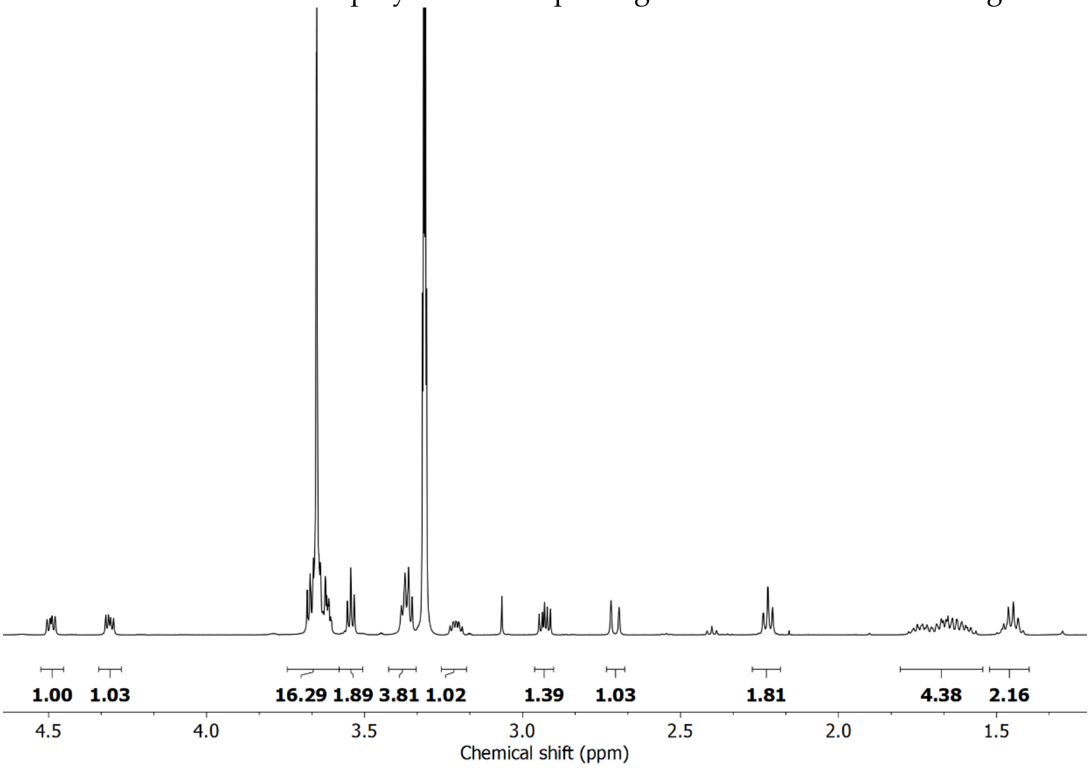

Figure S1. ^{1}H-NMR spectrum of compound **5**.

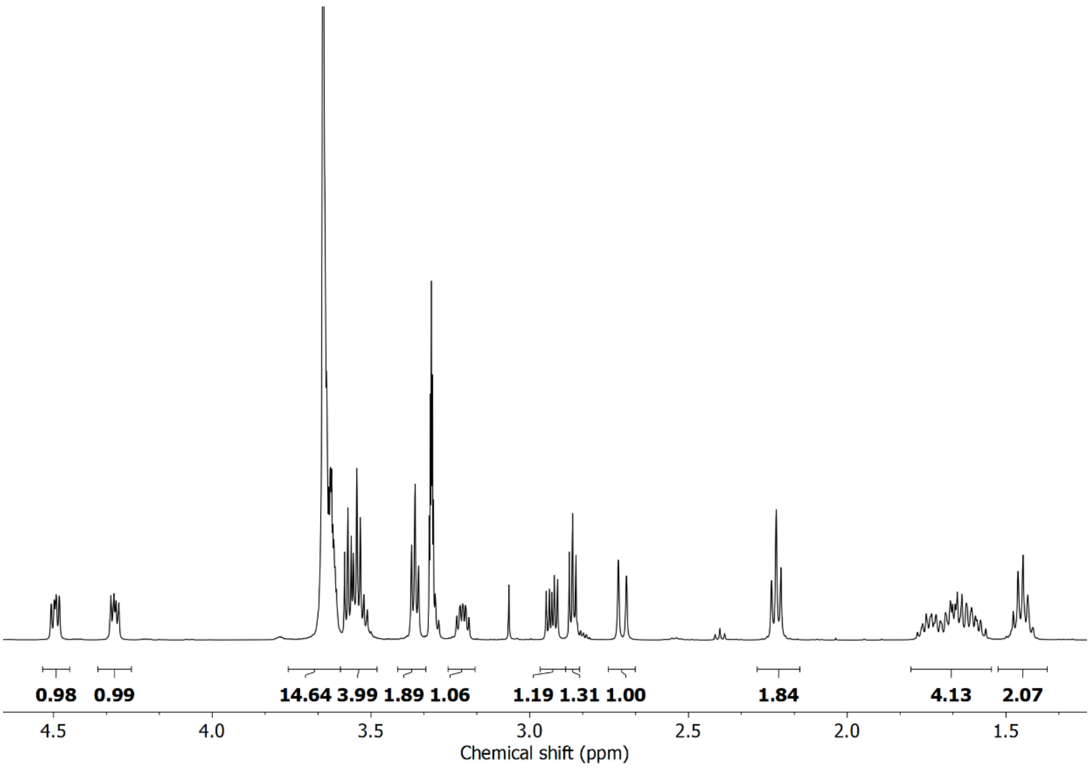

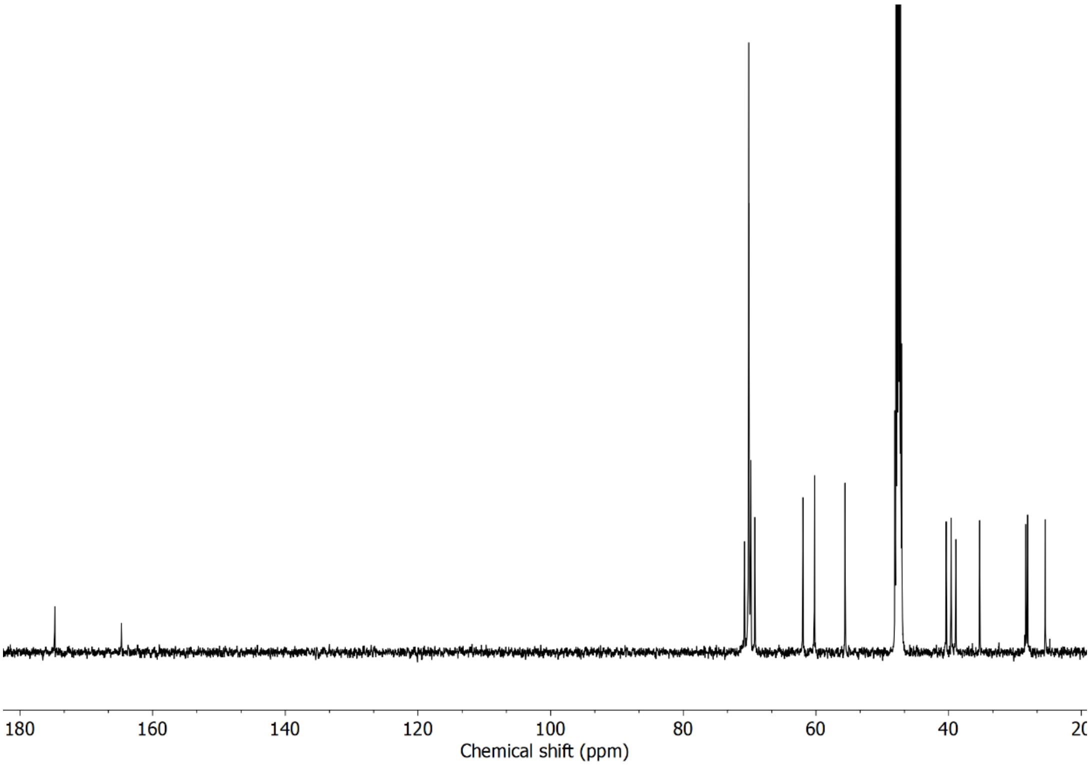

Figure S2. ^{1}H- (**top**) and ^{13}C-NMR (**bottom**) spectra of compound **6**.

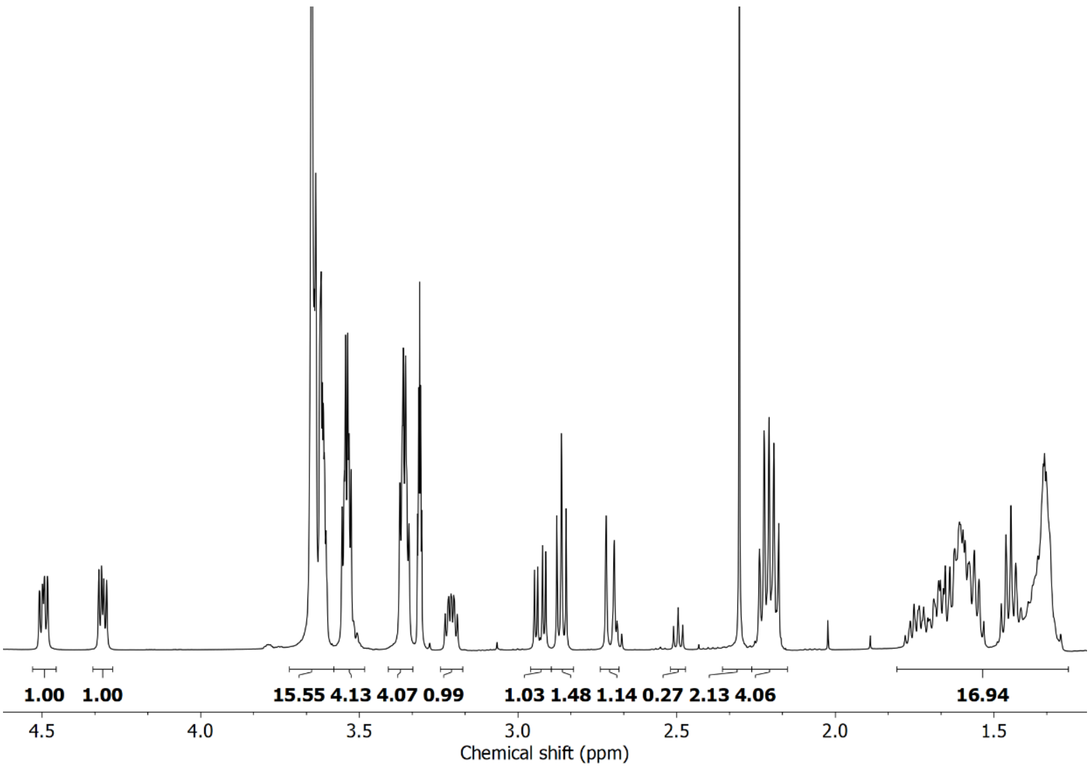

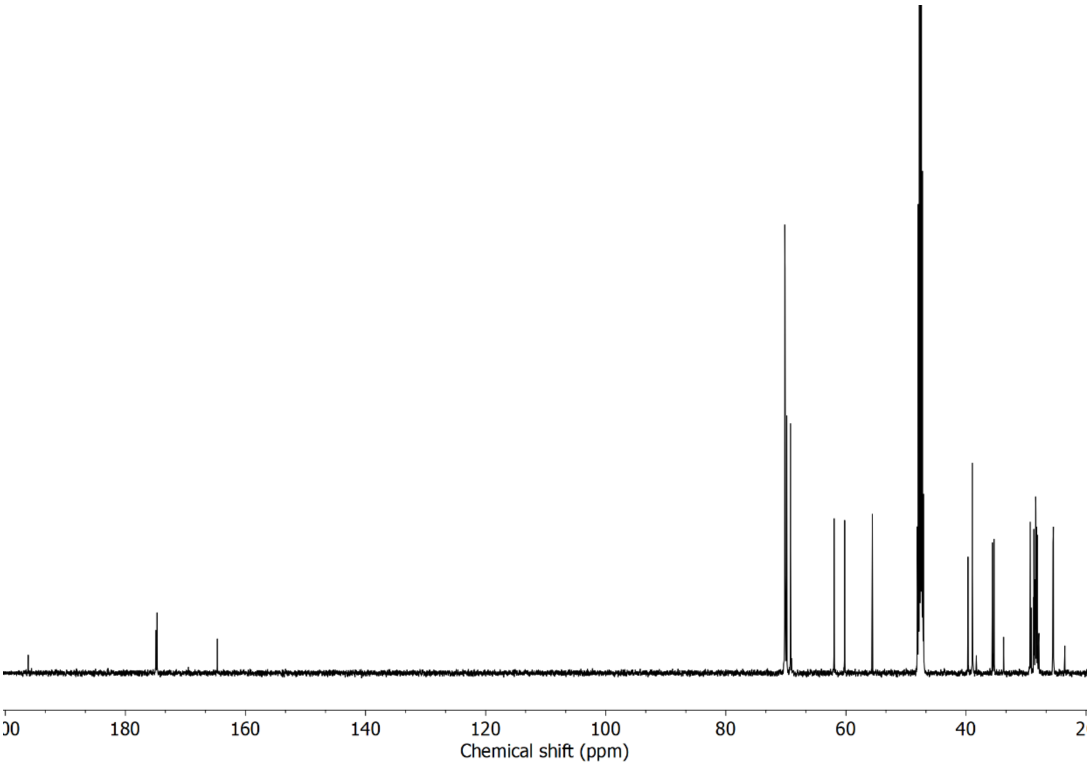

Figure S3. ^{1}H- (**top**) and ^{13}C-NMR (**bottom**) spectra of compound **7**.

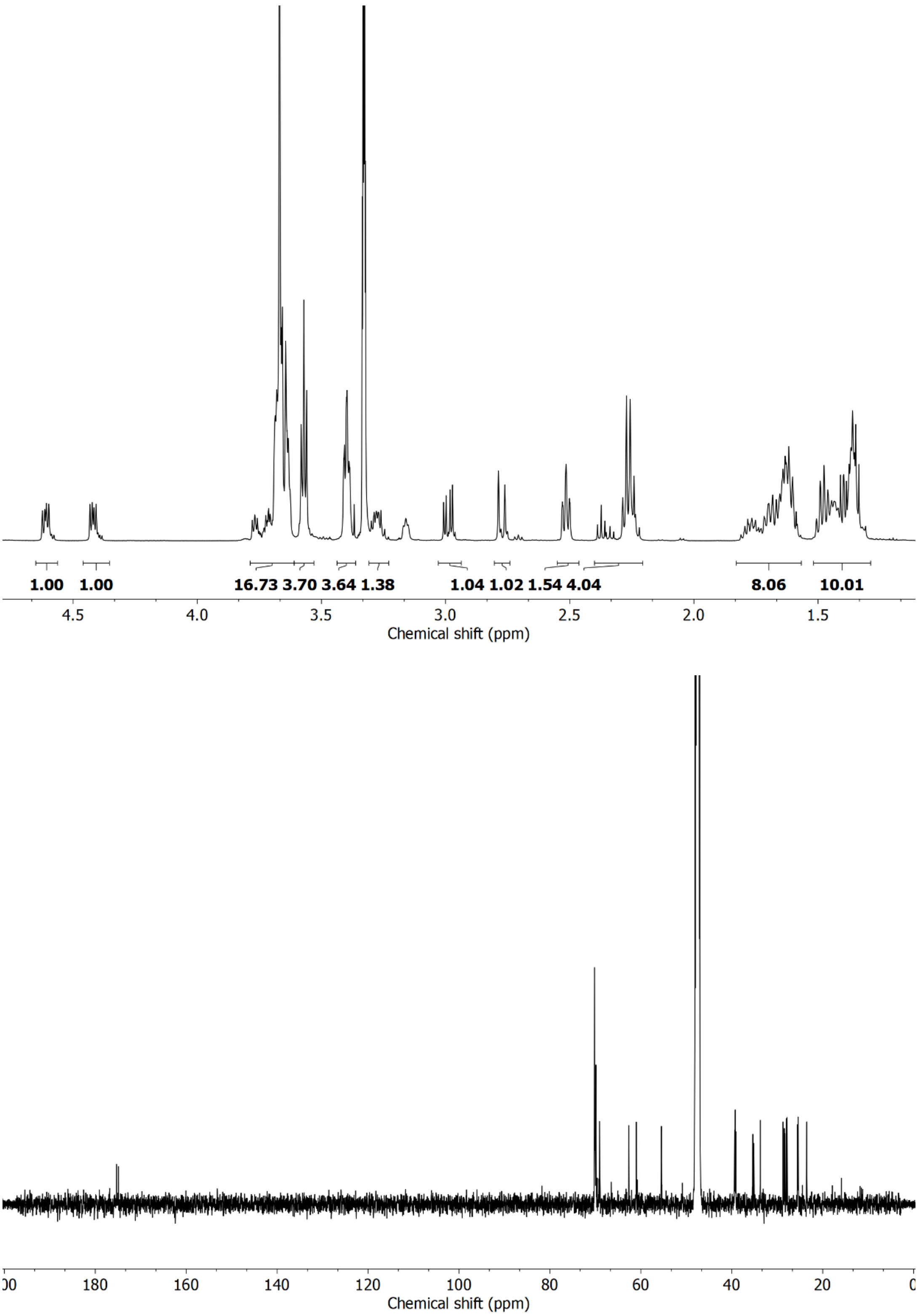

Figure S4. ^{1}H- (**top**) and ^{13}C-NMR (**bottom**) spectra of compound **1**.

2. Characterization of AuNPs

2.1. Gold Core

The NP sizes and dispersity were calculated by performing statistics (average, standard deviation) directly on the count of individual nanoparticles and not by fitting the histogram to any distribution (e.g. gaussian).

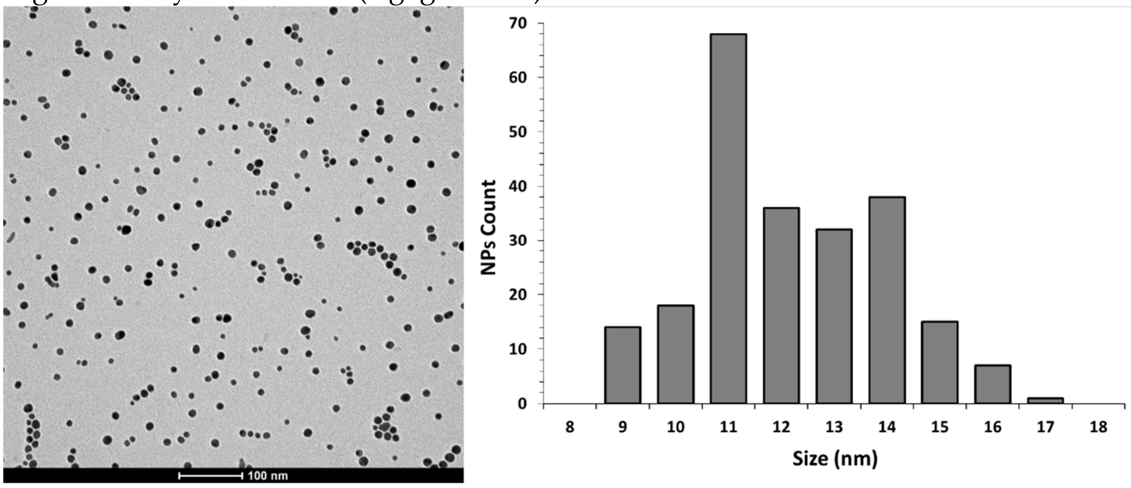

Figure S5. TEM image and histogram of the NPs analyzed for the size characterization of **AuNP1**.

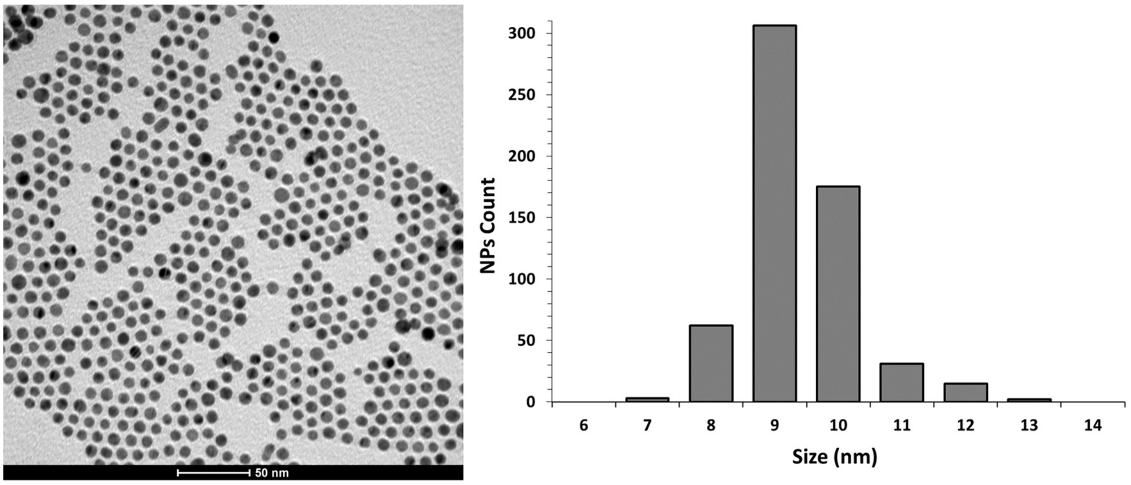

Figure S6. TEM image and histogram of the NPs analyzed for the size characterization of **AuNP2**.

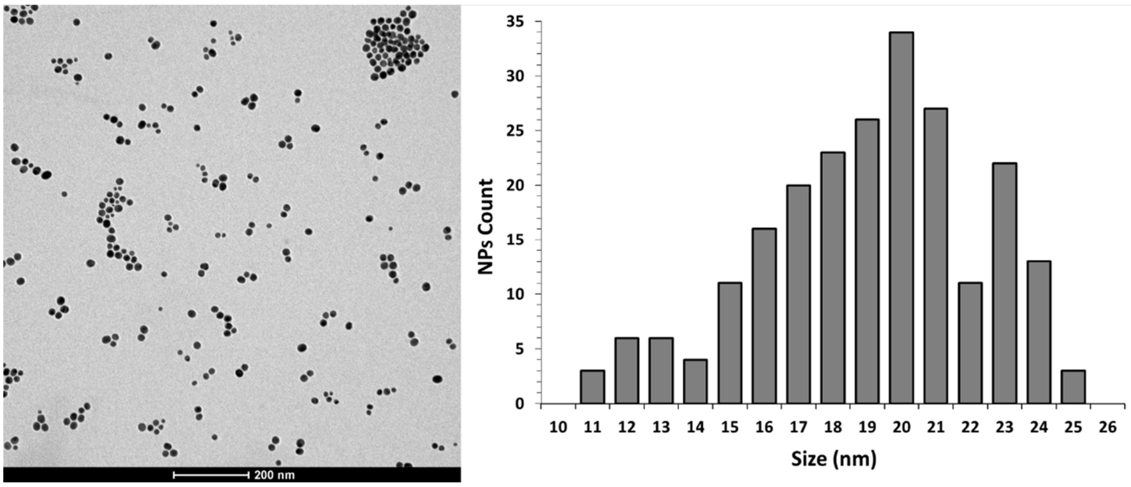

Figure S7. TEM image and histogram of the NPs analyzed for the size characterization of **AuNP3**.

2.2. Organic Monolayer

The amount of thiol per mass unit of nanoparticle can be easily calculated from the TGA data. Because all analysis are done before thiol exchange, the organic monolayer is composed only by thiol **2**. By dividing the organic mass fraction by the molecular weight of the thiol minus the hydrogen atom of the sulfhydryl group (368.49 $g \cdot mol^{-1}$), released when bound to gold, the desired value is directly obtained.

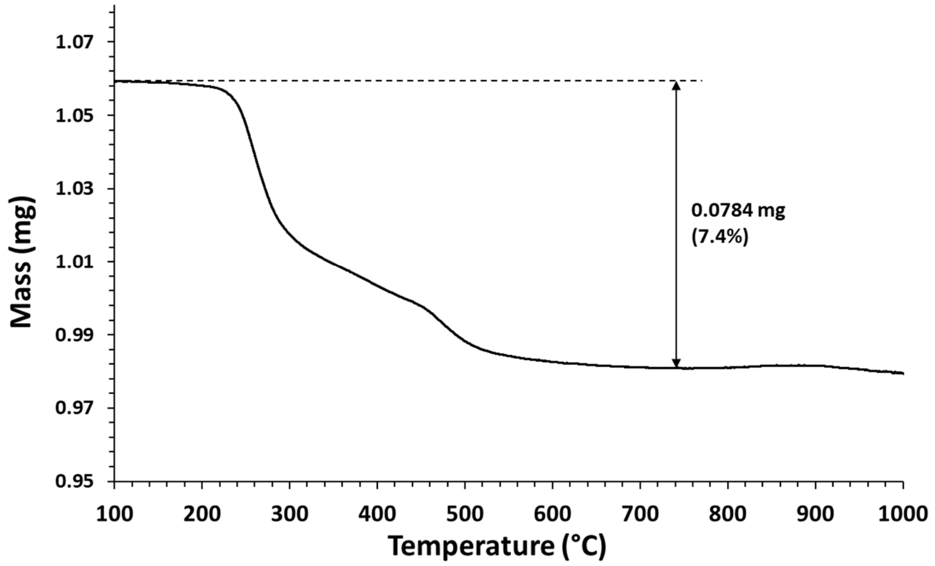

Figure S8. Thermogravimetric analysis of **AuNP1** before exchange with **1**.

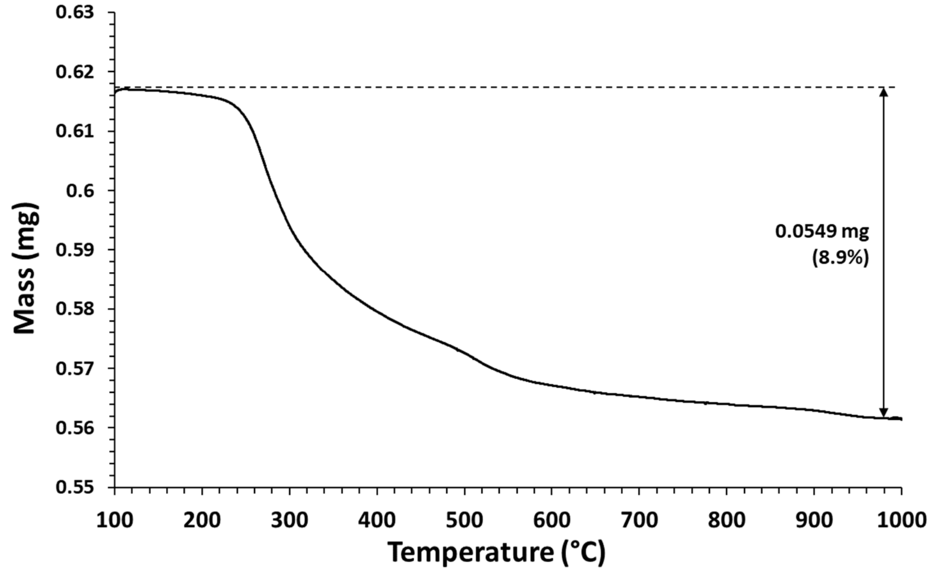

Figure S9. Thermogravimetric analysis of **AuNP2** before exchange with **1**.

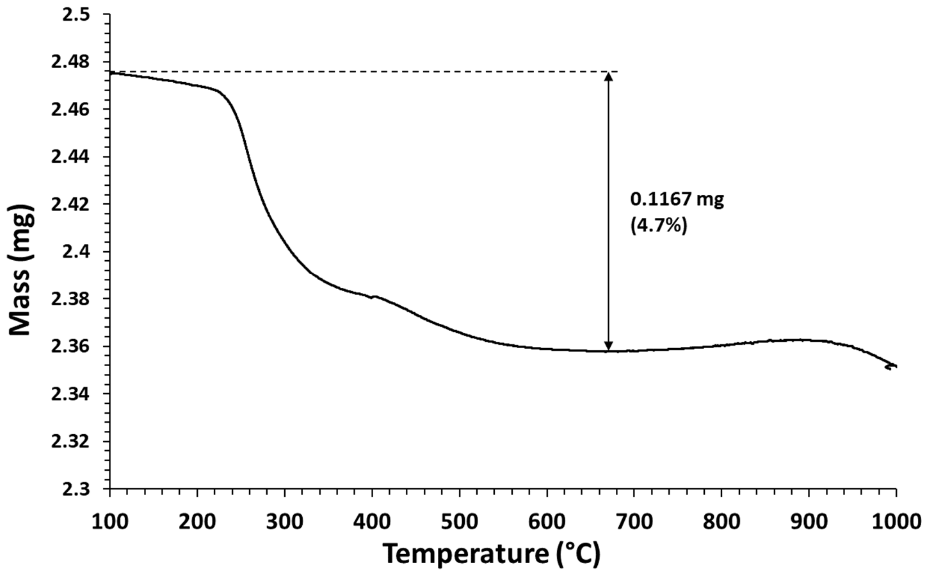

Figure S10. Thermogravimetric analysis of **AuNP3** before exchange with **1**.

3. Quantification of Thiol Exchange

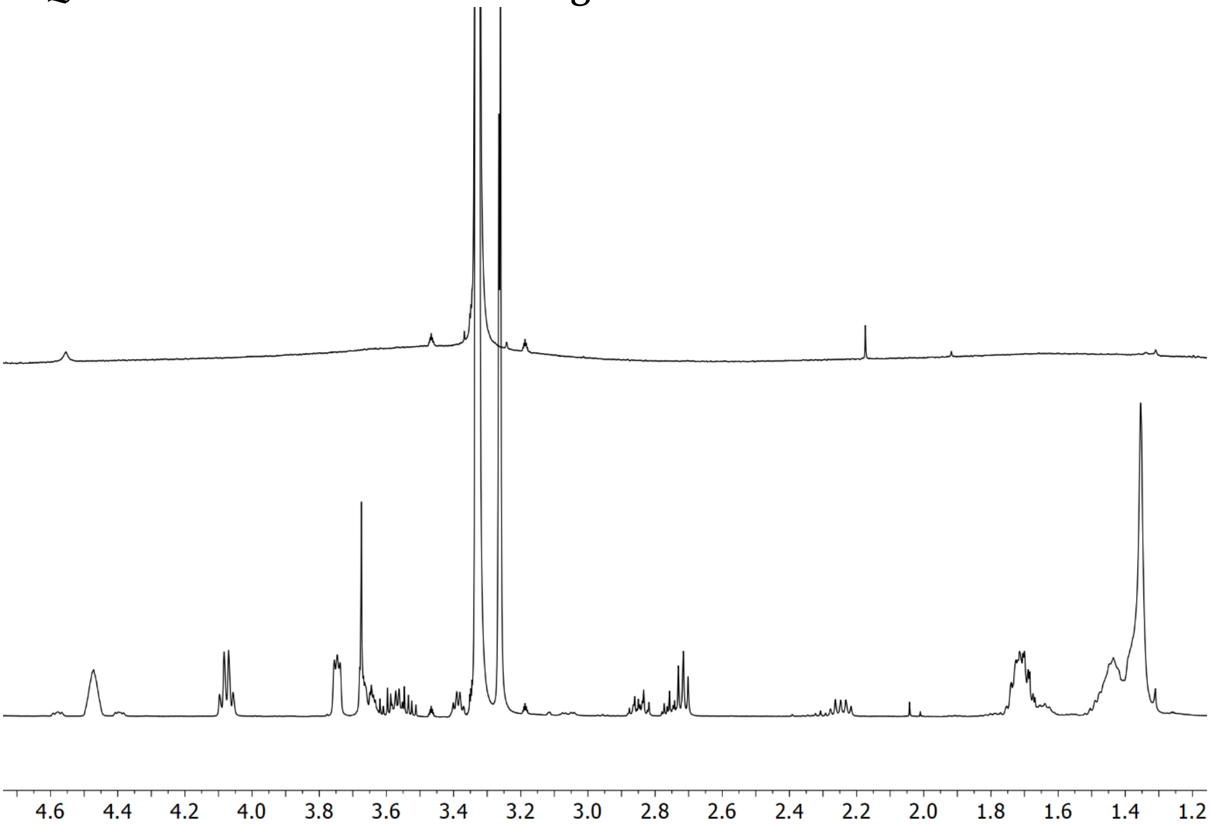

Figure S11. ^{1}H-NMR spectra of AuNP2 before (**top**) and after (**bottom**) the addition of iodine, same vertical scale employed. The lack of sharp thiol signals in the top spectra indicates the absence of free molecules in solution.

In order to determine the extent of the thiol exchange, the integral of two signals (one corresponding exclusively to each thiol) were compared. To minimize the error due to the low intensity of the signals of **1**, the average of two signals was employed. It must be taken into account that the signals selected for **1** corresponds to one single proton each, whereas that belonging to **2** corresponds to two equivalent protons.

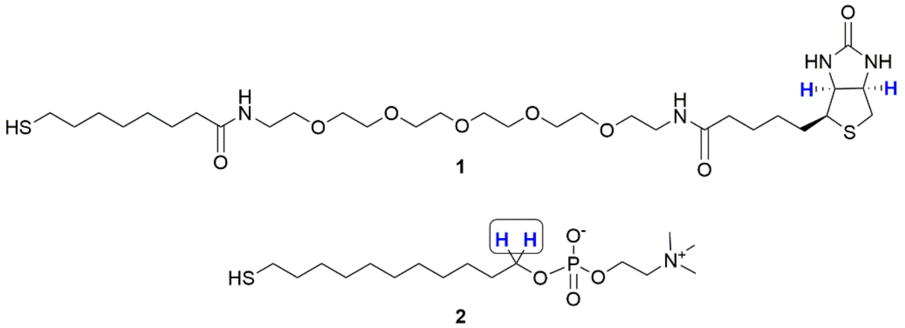

Scheme S2. Signals of thiols **1** and **2** employed for the quantification of the exchange. Notice that signals from **1** are not equivalent.

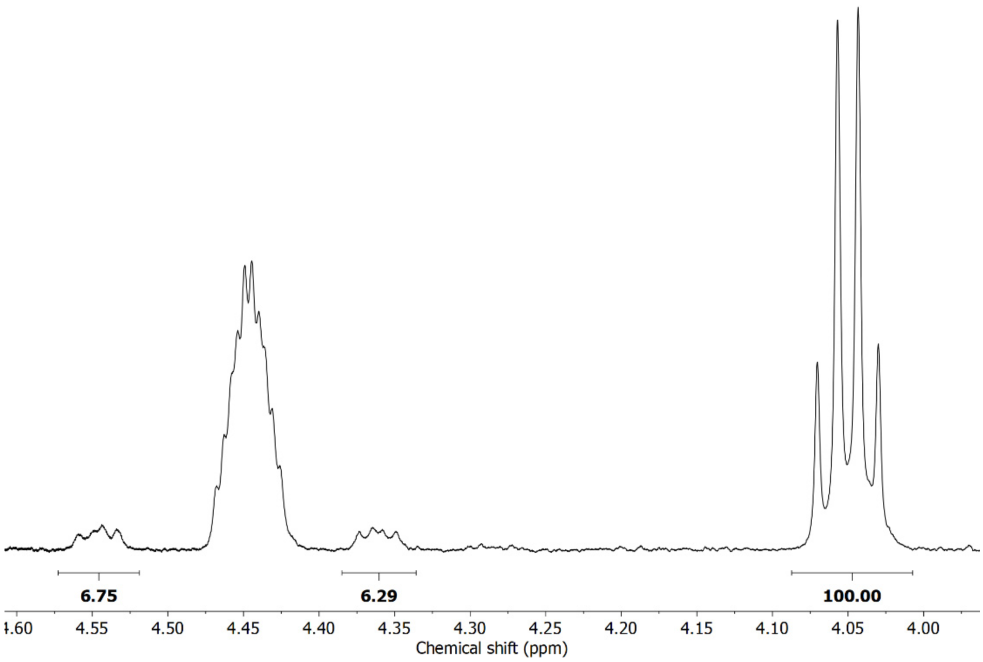

Figure S12. ¹H-NMR signals employed for the calculation of the % of biotin in **AuNP1**.

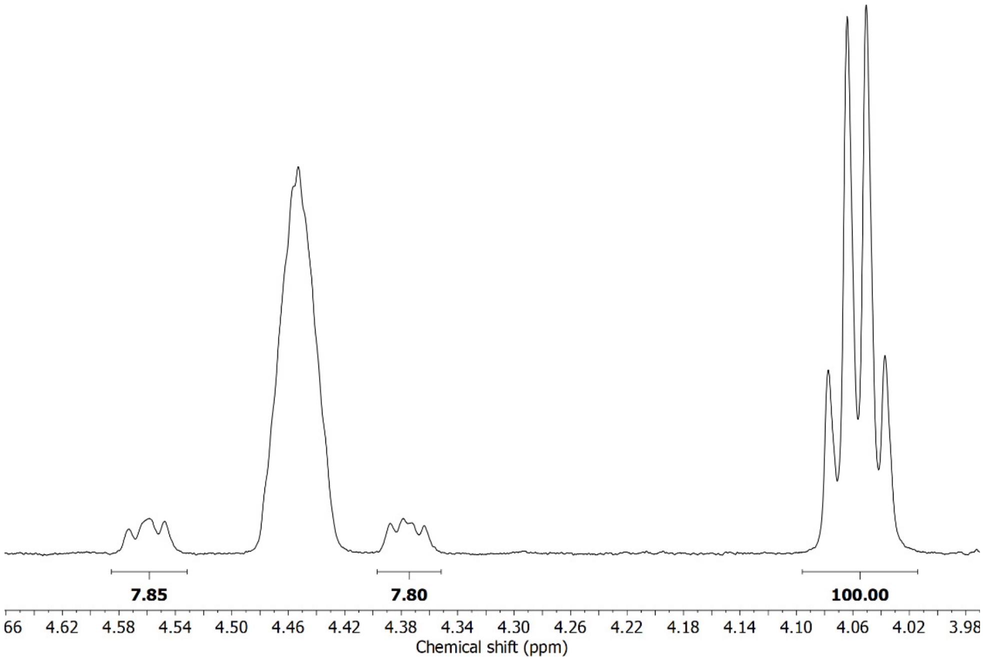

Figure S13. ¹H-NMR signals employed for the calculation of the % of biotin in **AuNP2**.

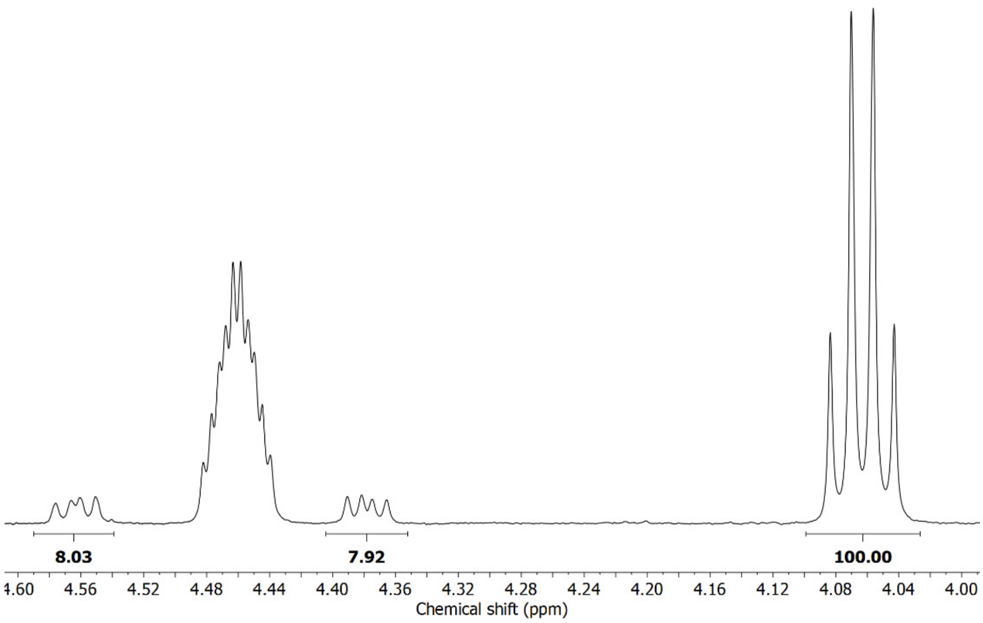

Figure S14. ^{1}H-NMR signals employed for the calculation of the % of biotin in **AuNP3**.

4. Assessment of Biotinylated AuNPs-Avidin Interaction

The gold nanoparticles were dissolved in buffer (HEPES 10 mM pH 7.3, 150 mM NaCl, 10 mM $MgCl_2$) and 4 equivalents of avidin (with respect to the active biotin thiols on the nanoparticles) were added and the solution left stirring for 1 hour. The excess of avidin was removed by filtrating over 100 kDa filters and washing with buffer 5 times.

In a fluorescence cuvette, the avidin covered gold nanoparticles are set to 0.8 absorbance at 520 nm and then the dye (Atto 565, stock solution 0.29 mM in DMSO) is added directly to the cuvette. The cuvette is shaken, allowed to incubate for 1–2min and the fluorescence measured (Exc 565 nm, Em 590 nm). This process is repeated until no decrease of the fluorescence between two consecutive measurements is observed. The process is repeated iteratively until the titration has concluded.

The control experiment was made in a completely analogous way but adding 5 µM of biotin to the cuvette.

The concentration of the gold nanoparticles was obtained from its absorbance and calculated extinction according to reported methods [1].

Results of the titration experiments for the two nanoparticles tested can be observed below in Table S1.

Table S1. Results of the fluorescence titration experiments.

Sample	AuNP diameter (nm)	AuNP ε ($M^{-1}\cdot cm^{-1}$)	[AuNP] (nM)	$[Atto]_{saturation}$ (nM)	Avidin/NP (2 Atto/avidin)
**AuNP1**	11.7	1.74×10^8	4.60	106.4	11.6
**AuNP3**	18.6	8.11×10^8	0.99	93.9	47.6

4.1. TEM Micrographs

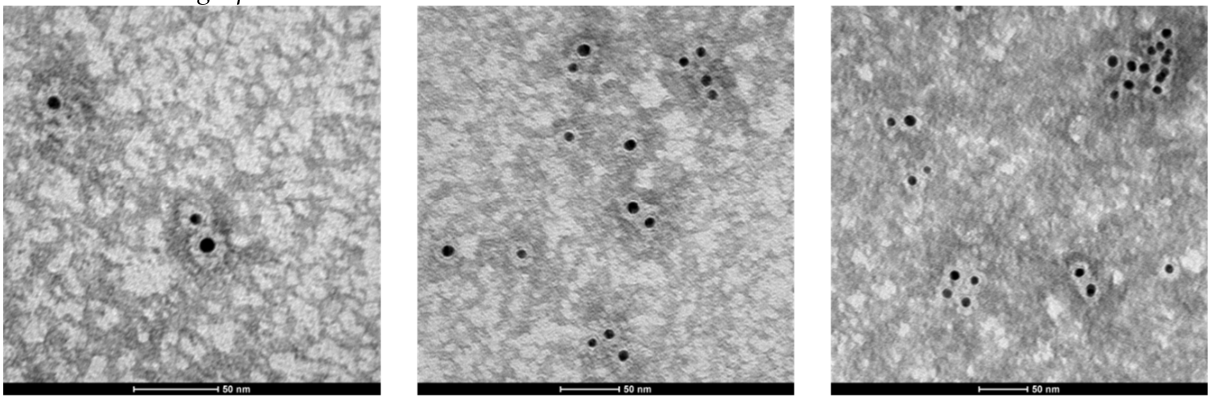

Figure S15. Selected TEM images stained with uranyl acetate. The light rings around the NPs correspond the avidin corona.

5. Avidin-AuNPs Crosslinking

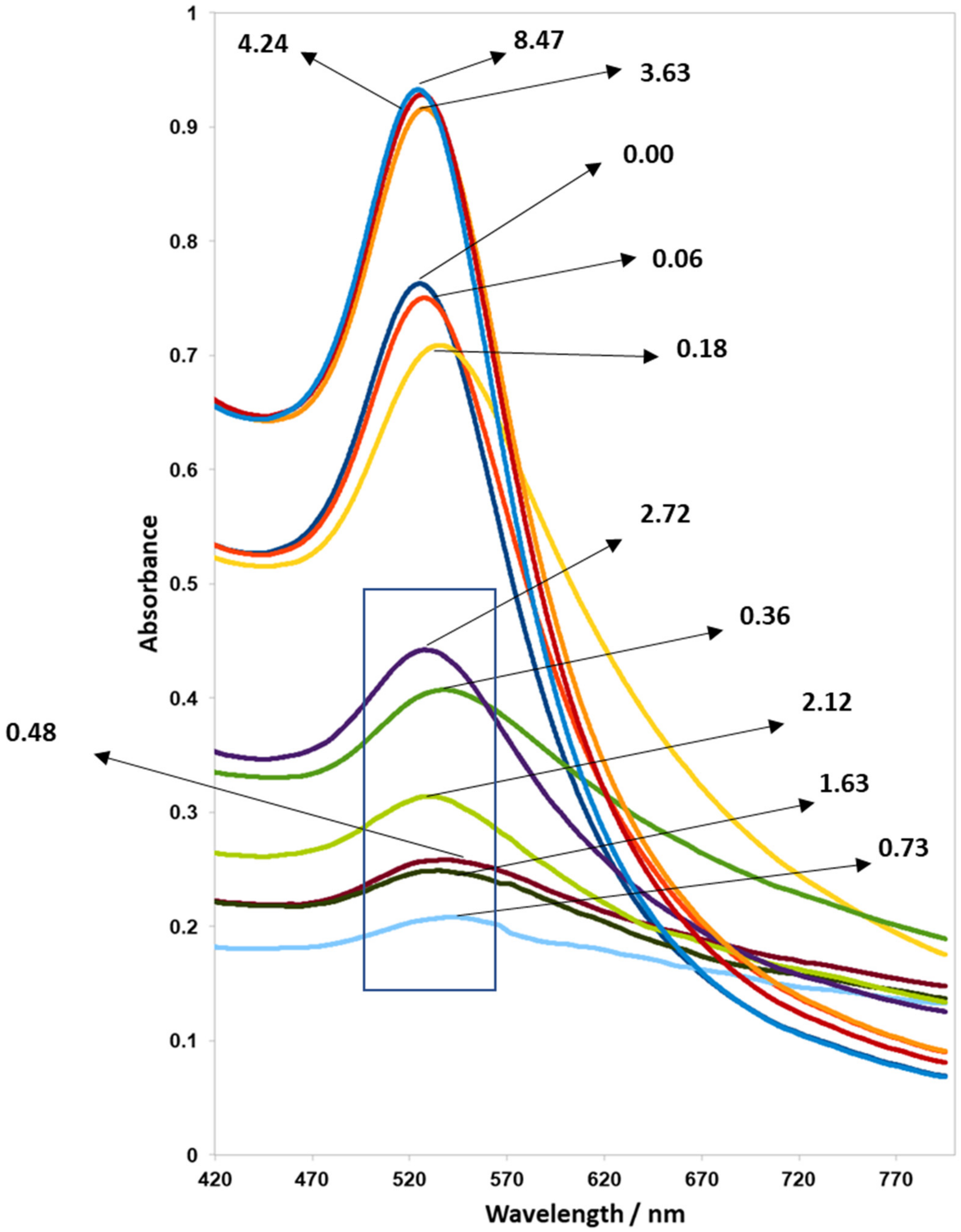

Figure S16. UV-Vis spectra of the cuvettes shown in Figure S16 and 5B. The arrows indicate the equivalents of avidin added with respect to the biotin available for binding. The rectangle on the graph includes spectra with samples showing the formation of precipitates (which is extensive for samples from 0.48 to 2.12).

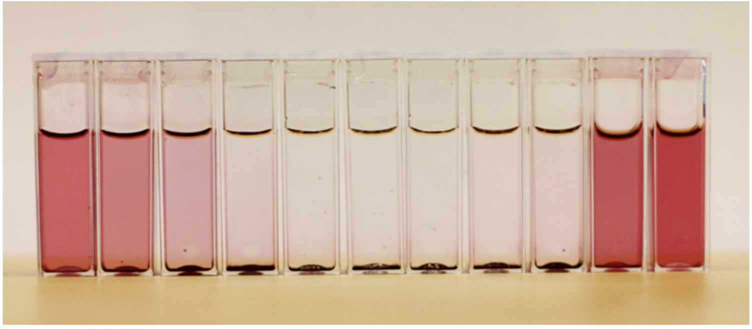

Figure S17. Pictures of the cuvettes shown in Figure 5B when allowed to deposit, precipitation is clearly visible in those where crosslinking has occurred.

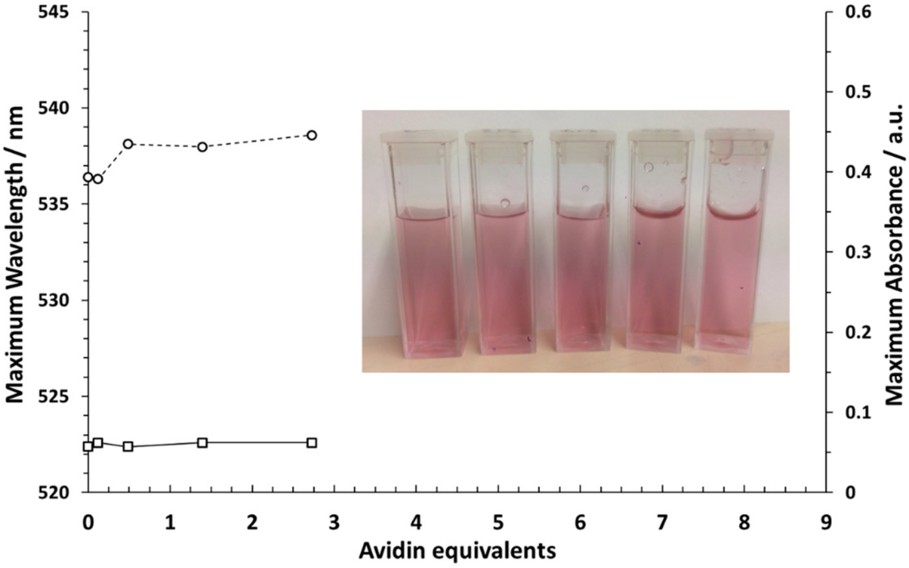

Figure S18. Position of the maximum of the SPR band (left axis, squares) and its absorbance intensity (right axis, circles) of non-biotinylated AuNP2 (prior to thiol substitution with **1**) upon addition of increasing amounts of avidin. The same scale of Figure 5 has been used for comparison. Inlet: Picture of the cuvettes, in order, showing non perceptible changes.

References

1. Liu, X.; Atwater, M.; Wang, J.; Huo, Q. Extinction Coefficient of Gold Nanoparticles with Different Sizes and Different Capping Ligands. *Colloids Surf. B Biointerfaces* **2007**, *58*, 3–7.
